# Advancing cell therapy manufacturing: an image-based solution for accurate confluency estimation

**DOI:** 10.3389/fbioe.2025.1651144

**Published:** 2025-10-01

**Authors:** John Mason, Martin Kraft, Jakob Hueckels, Hao Wei, Konstantinos Spetsieris

**Affiliations:** ^1^ CMC Digital Transformation and Data Science, Bayer, Berkeley, CA, United States; ^2^ CMC Process Engineering and Technology, Bayer, Wuppertal, Germany; ^3^ CMC Digital Transformation and Data Science, Bayer, Wuppertal, Germany

**Keywords:** cell therapy, imaging, PAT, data science, cell culture, manufacturing

## Abstract

Cell therapies represent a transformative approach for treating diseases resistant to conventional therapies, yet their development and manufacturing face significant hurdles within the biopharmaceutical sector. A critical parameter in the production of these therapies is cell confluency, which serves as both an indicator of biomass in adherent cultures and a determinant of product quality. However, existing methods for measuring confluency are often inadequate for the large-scale cultivation systems used in industry, and current software solutions lack comprehensive automation capabilities necessary for a manufacturing environment. This article introduces a novel image-based software application designed for accurate cell confluency estimation, integrated with a high-throughput microscopy system. Utilizing a machine-learning model for pixel classification, the application facilitates efficient image and metadata processing in a cloud environment, delivering results through an interactive web interface. By incorporating methods from process analytical technologies, manufacturing data digitalization, and data science, this platform enables automated image acquisition, storage, analysis, and reporting in near-real time. The proposed solution aims to streamline the manufacturing process of cell therapeutics, ultimately enhancing the reliability and speed of delivering these innovative treatments to patients.

## 1 Introduction

Cell therapies (CTs) represent a transformative approach to treating diseases that are resistant to traditional therapies (Hoang et al., 2022; O'Brien and Barry, 2009; Yamanaka, 2020). Despite their immense potential, the biopharmaceutical industry faces significant challenges in the development and commercial manufacturing of these therapies, primarily due to the complexities of using living cells as therapeutic agents. One critical aspect of cell therapy manufacturing is cell confluency (the extent of cell coverage in a culture dish), which serves as a proxy for biomass in adherent cultivation vessels and is essential for ensuring the quality of intermediate cell products. Accurate measurement of cell confluency is vital to avoid growth inhibition, minimize lag phases in subsequent cultivations, and facilitate timely harvesting through data-driven decisions.

However, current methods for estimating confluency are often unsuitable for the large, stacked cultivation vessels commonly employed in industrial production. Existing software solutions frequently lack the necessary end-to-end automation for data collection, modeling, analysis, and display, particularly in regulated environments adhering to Good Manufacturing Practices (GMP). Furthermore, traditional manufacturing methods struggle to monitor critical biological parameters effectively, as equipment and analytics are often retrofitted rather than specifically designed for cell therapies (Bashor et al., 2022; Lipsitz et al., 2016).

To address these challenges, the development of new Process Analytical Technologies (PAT) is essential. These technologies can enhance real-time process understanding during both development and commercial production, thereby reducing variability and the risk of batch failure (U.S. Food and Drug Administration, 2004; Abraham et al., 2018; Wang et al., 2021). However, integrating in-line or on-line PAT sensors remains problematic due to the variety of cellular products and cultivation vessels, which can hinder sensor access and increase sensitivity to analytical perturbations. Conventional bulk analytical methods often fall short in providing insights into non-homogeneous cell populations.

Recent advancements in imaging technologies and machine learning (ML) present promising solutions to these analytical challenges. These tools offer versatility, minimal biological interference, and the capability for single-cell or aggregate resolution (Berg et al., 2019; Kan, 2017). As mentioned above, one significant gap in current analytics is the readout for biomass production in adherent cultivation vessels, where cell confluency is widely used as a proxy. The CM incubation monitoring device from Evident (formerly Olympus) (Mizunaka, 2020) has been identified as a suitable imaging solution for monitoring cell confluency in large, stacked vessels without relying on contrast-enhancing methods.

To meet the pharmaceutical industry’s requirements for routine confluency estimation, an automated solution that integrates image acquisition, storage, pre-processing, analysis, and reporting in near-real time is necessary. While several software packages, such as Cell Profiler (Carpenter et al., 2006) and ImageJ (Schneider et al., 2012), facilitate automated image analysis workflows in laboratory settings (Jaccard et al., 2014; Logan et al., 2016), they often fall short of the comprehensive data engineering and analysis systems required for regulated industrial environments. These existing tools provide valuable functionalities for laboratory applications; however, industrial-level applications demand additional components such as cloud infrastructure, extensive automation and integration of multiple systems and process, and their rigorous validation to ensure compliance and scalability.

In this article, an image-based software application for cell confluency estimation, integrated with a high-throughput microscopy system is discussed. This industry-level platform was developed through the integration of PAT, digitalization, data engineering and data science technologies. The application employs a machine learning model for pixel classification, enabling efficient data processing and analysis. The authors believe that such platform technologies can significantly streamline the development and commercial manufacturing of cell therapeutics, thereby addressing the current challenges and positively contributing to faster and consistent delivery of promising CT treatments to patients.

## 2 Materials and methods

### 2.1 Software application overview

To streamline the development and commercial production of cell therapeutics, the image-based software application for cell confluency estimation comprises several key functional components. These include image data acquisition, automated data transfer and storage, data modeling and analysis, and result reporting.

**FIGURE 1:**
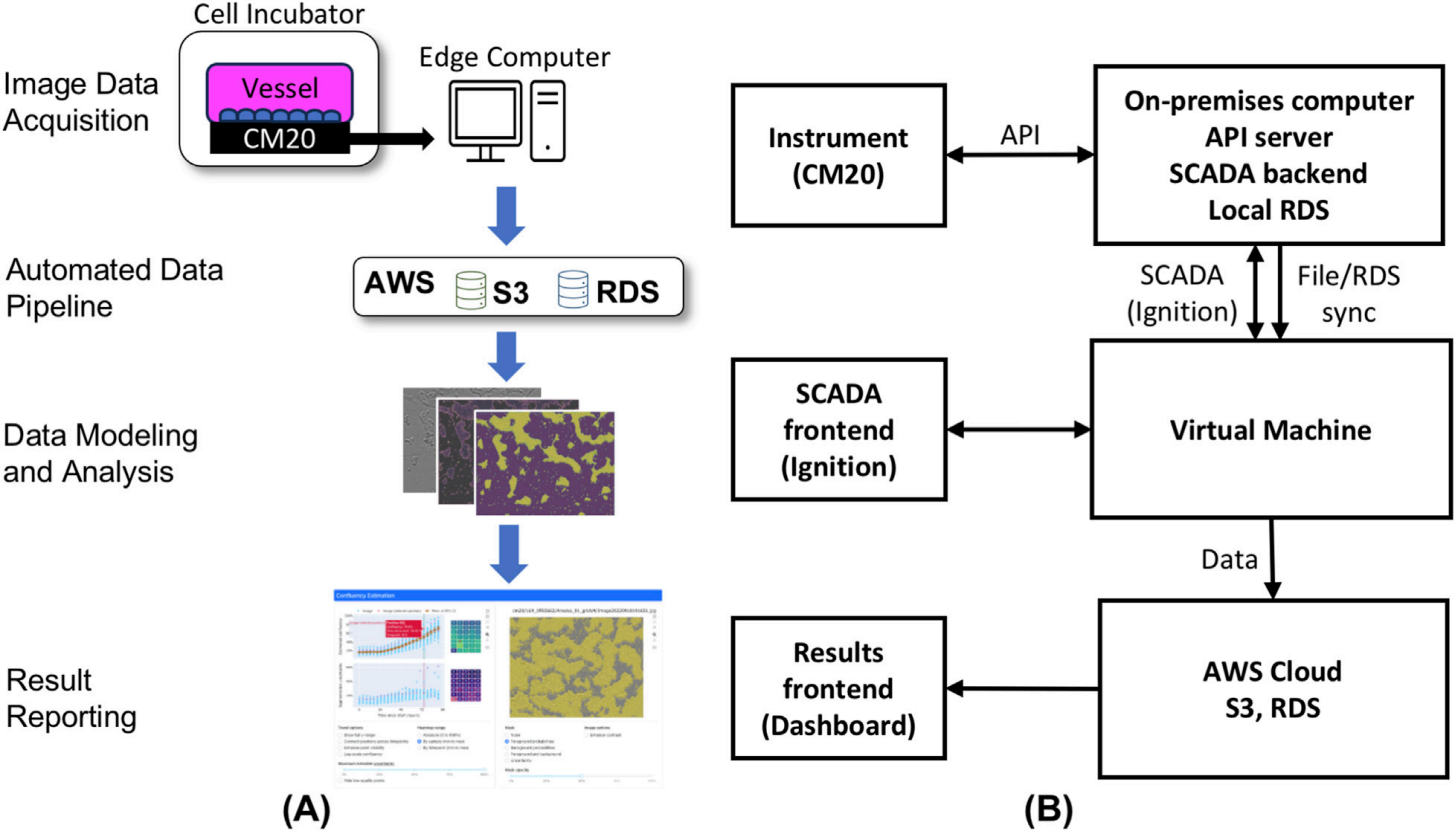
**(A)** Schematic overview of confluency estimation software application. The application comprises image data acquisition, automated data pipeline, data modeling and analysis and results reporting. **(B)** Network data pipeline from instrument to cloud application. The first layer features a USB-connected instrument linked to an on-premises computer running containerized applications (Ignition, RDS, API-server). The second layer includes the SCADA front-end (Ignition) that commands the instrument via an API and transfers image files and metadata to AWS RDS and S3, respectively. Processed images and metadata are displayed in an AWS-hosted cloud application.

As depicted in Figure 1A, the image data acquisition stands as the initial unit within the integrated application framework. Specifically, a high-throughput image capturing device (CM20 incubation monitoring system (Mizunaka, 2020)) is employed to automatically capture images of cells growing within the cell culture vessel.

Subsequently, as part of the automated data transfer and storage pipeline, images and metadata are extracted from the on-premises device associated with the image capturing instrument and then transferred to a cloud-based Simple Storage Service (S3) (Amazon S3, 2017) solution from Amazon Web Services (AWS) (Amazon Web Services, 2011) for storage.

For data processing and analysis, the acquired image and metadata undergo preprocessing. Following this, a machine learning model, trained to estimate cell confluency through image pixel classification, is utilized to analyze the processed image data and predict cell confluency and store the analysis results into a relational database for subsequent display.

Finally, the predicted cell confluency results, along with other relevant quality metrics, are presented through an interactive web-based interface, implemented using Dash (Parmer et al. 2024) for Python (2002). The specific details of the aforementioned functional components are further discussed in subsequent sections.

### 2.2 Cell cultivation and monitoring

Human induced pluripotent stem cells (hiPSCs, episomal) from Gibco™ (A18945) were grown in Essential 8™ medium (Gibco™, A1517001) in TC-treated either CellSTACK® (CS) culture vessels with 1-5 chamber layers (Corning®, 3,268), Nunc™ Cell Factory™ (CF) culture vessels with 1-4 chamber layers (Thermo Scientific, 140004), or T-225 flasks (Corning®, 431082) all coated with human recombinant laminin 521 (BioLamina, LN521). Cultures were incubated at 37 °C in a humidified atmosphere of 5% CO2 and passaged weekly with TrypLE™ Express (Gibco™, 12604013) for dissociation and ROCK inhibitor Y-27632 (Sigma-Aldrich, SCM075) to prevent apoptosis within the first 24 h after seeding. Medium was exchanged every second day, starting 1 day after seeding. The number of passages was kept below 30.

Automated microscopy systems from Evident/Olympus (Provi CM20) (Mizunaka, 2020) inside the incubator were used for constant monitoring. Each CM20 monitoring platform (hereinafter referred to as head) was connected to a compact PC (Lenovo, M920 Tiny) and controlled via the CM20H API (version 1.1.1). An image acquisition protocol (API-script) was created to acquire images of 2048x1536 pixel from 35 positions as an equally spaced 5x7 grid within the observation window of the CM20 heads. The autofocus function was used to find the optimal focus plane for each position. LEDs for sample illumination were switched when necessary (reaching boundaries of the observation window), default exposure times were used.

Cultivation vessels were placed onto the CM20 heads in a way to ensure representative monitoring of the growth area as well as a leveled surface to avoid inhomogeneous cell or medium distribution. A cycle of the API-script was performed with an interval of 4 h, starting within an hour after seeding until end of cultivation.

### 2.3 Image acquisition and data processing pipeline

The image acquisition process, shown in Figure 1B comprises two main components: the containerized backend and the frontend. The backend system is orchestrated through an on-premises computer that interfaces with the imaging instrument via a USB connection. This on-premises computer hosts an API-server software that facilitates communication through the Representational State Transfer (REST) protocol. In tandem, a SCADA system, specifically Ignition, orchestrates the data flow between the instrument, the backend API-server, and the AWS cloud Relational Database Service (RDS) (Amazon Relational Database Service, 2014) and S3 buckets.

Operators are granted the capability to select and initiate protocols, assigning specific names, and setting parameters such as interval frequency and overall duration. Cell culture imaging typically occurs at 4-h intervals over the course of a week. Operators also have the flexibility to pause and resume runs, e.g., to perform tasks such as media exchange.

Within the backend, an Ignition script engine is employed to load predefined procedures, execute the corresponding commands, and relay them to the imaging instrument via the REST API. Post-image capture, the files are first stored locally, along with associated metadata in a local RDS. These metadata encompass details such as the sample date, AWS S3 storage location, filename, information about the procedure protocol, capture positions, and specific instrument data. Subsequently the data is transferred for storage to AWS cloud RDS and S3.

### 2.4 Data analysis and integration

A scheduled task continuously monitors the RDS for new images awaiting analysis. These images, fetched from their AWS S3 buckets, are subjected to analysis using the confluency estimation model, details of which are elaborated in subsequent sections of this manuscript. The model’s output, including confluency metrics and other relevant statistics, is then recorded back into the RDS. This integration allows for real-time visualization through a dedicated dashboard.

### 2.5 Labeling approach

Image pixels were labeled as either “foreground” or “background” based on criteria determined in concert with subject matter experts. The “background” represents the empty portion of the visual field, whereas the “foreground” represents cells and other cell-like particles. Ambiguous regions (not identifiably “foreground” or “background”) were intentionally not labeled.

### 2.6 Training data

To provide high-quality training data for model training, one image was selected from each of 23 timepoints from a 90 h culture of hiPSCs in a CS culture vessel, for a total of 23 images. This allowed for the model to capture a diversity in cell morphology from the time of seeding out to nearly 100% confluency. At each timepoint, an image at a random position (from a total of 35) was selected to capture additional variability. The intention of this strategy is to provide the model with a training data set that is richly informed by the potential variability in the images, possibly due to factors such as changes in illumination, condensation or defects on the plate, and other difficult-to-control factors. In total, only 143k pixels were labeled for training, less than 0.20% of the pixels in the 23 selected images. In the results, we will demonstrate that the model can achieve high accuracy with relatively little training data.

### 2.7 Test data

To assess model generalizability, a set of test labels was annotated for a separate hiPSC cultivation run across three different cell cultivation vessels (T-225, CellFactory (CF)1, and CF4) and over three nominal confluency ranges (low, med, and high, or 0%–33%, 34%–66%, and 67%–100%). In total, 16.8M pixels from 93 images were labeled for testing, comprising 11.7M foreground and 5.1M background pixels. Subtotals are provided in the Supplementary Table 1.

### 2.8 Image preprocessing

Prior to classification, every image is subjected to a bank of predefined filters. As with other techniques (Berg et al., 2019; Arganda-Carreras et al., 2017) the filters capture a diversity of features assessed across multiple scales. Broadly speaking, these filters extract features such as intensity (simple Gaussian blur), gradient (gradient magnitude of the Gaussian), edge/peak strength (Hessian eigenvalues and the Laplacian of the Gaussian), and texture (structure tensor eigenvalues). The stack of filter outputs is transformed into a matrix in which each row corresponds to a pixel coordinate in a particular image, and each column corresponds to a filter applied at some scale (a blur radius of 0.5, 1, 2, 4, or eight pixels). See Supplementary Material, Section 3.1 for additional details.

### 2.9 Modeling approach

Confluency estimation is based on image segmentation to determine the “foreground” and “background” parts of each image. Traditional image segmentation methods, such as Otsu thresholding (Otsu, 1979), exhibit significant limitations in the context of complex images like brightfield microscopy of cell colonies. These methods primarily depend on pixel intensity without consideration to the spatial relationships between pixels, an aspect that is essential for achieving accurate segmentation in images featuring complex structures. Additionally, it operates under the assumption of a clear, bimodal histogram to differentiate between foreground and background. However, this assumption often fails in real-world scenarios where low contrast, uneven lighting, and overlapping structures are prevalent. Otsu’s method is also inherently sensitive to noise and artifacts, which can lead to inaccurate and unreliable segmentation outcomes. In contrast, an approach that utilizes traditional image filters (e.g., Laplacian) to generate features in conjunction with a traditional machine learning model offers a substantial advantage. By extracting these features and employing a traditional machine learning algorithm for pixel classification, this method captures complex patterns and relationships that traditional segmentation methods overlook. This capability allows for a more robust framework for semantic segmentation, resulting in significantly enhanced accuracy.

In this context, the confluency estimation task can be formulated as a binary classification problem in which each pixel is classified as either “foreground” or “background”. A machine learning model for classification was employed for this task. Specifically, a random forest classifier (RFC) was used to predict the label of each class from the features extracted for each pixel coordinate using image filters (as explained in previous section). The default settings for scikit-learn’s implementation were used, except for the maximum tree depth, which was restricted to 20 based on a preliminary evaluation of the trade-off between tree depth and accuracy. After training, the model was serialized (via Python’s pickle module) and uploaded to an S3 bucket for later retrieval during evaluation. See Supplementary Table 1, Section 3 for additional details.

### 2.10 Additional calculations

Following evaluation of the classifier, the confluency for a given image is calculated as the fraction of pixels classified as foreground. We further define the uncertainty for a given predicted pixel label as the entropy of the classification “probabilities”, i.e., the fraction of decision trees in the forest that assigns a pixel to a given class. For two classes, the base-2 entropy gives us uncertainty values ranging from zero (total agreement across all decision tree classifiers in the random forest ensemble) to one (even split between foreground and background predictions). The uncertainty for an image is then defined as the average uncertainty across all pixels.

### 2.11 Application testing

Documented user requirements were used to identify desired dashboard behaviors, which in turn were tested by simulating user interactions in an isolated environment with controlled data. Testing was automated via Pytest (2012). Separate (Docker, 2022) containers were utilized for serving the Dash (Parmer et al., 2024, Python, 2002) application and simulating dashboard interactions via Selenium, (2005) (facilitated by the Dash testing extensions). Additional containers were used to create controlled testing image sets using LocalStack, (2001) AND Amazon S3, (2017) and metadata using a containerized PostgreSQL (PostgreSQL, 2016) database. Graphical outputs of tests, captured using Selenium, were validated against references using the Pytest-Regressions extension (Pytest-Regressions, 2015). Testing of the confluency estimation model also utilized Pytest and extensions.

## 3 Results

To enable consistent cell therapy manufacturing, a data science application comprising an image analysis model and a dashboard was developed to assess confluency throughout the course of cell expansion in adherent cell culture.

### 3.1 Modeling results

**FIGURE 2:**
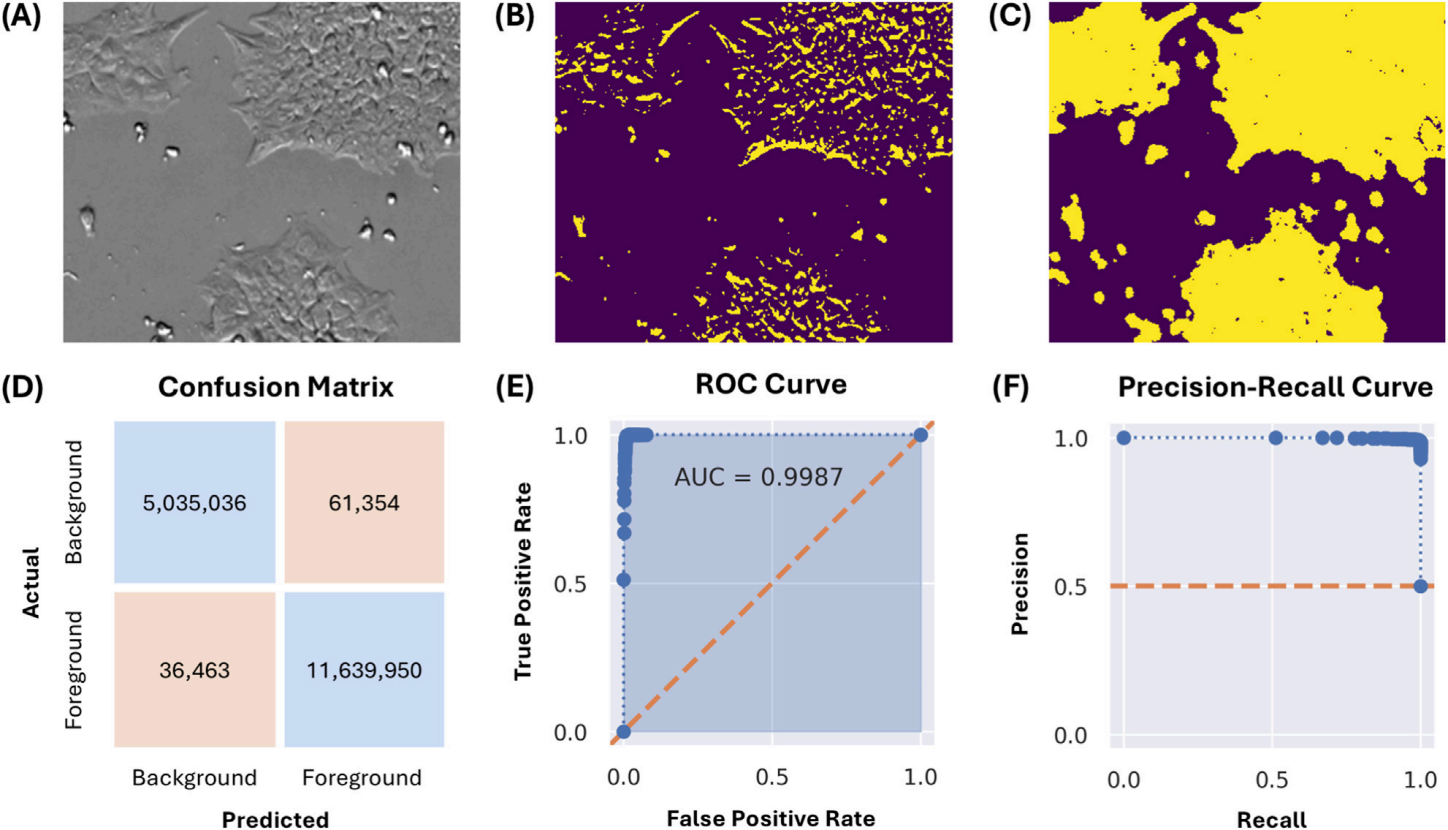
**(A)** A 250x300 pixel image subregion of a culture at approximately 50% confluency. **(B)** Image segmentation by Otsu thresholding. **(C)** Image segmentation using the described pixel classification approach. **(D)** Confusion matrix for the classification model evaluated on the test set, in number of pixels classified. **(E,F)** Receiver-operator characteristic (ROC) and precision-recall curves for the classification model evaluated on the test set, with decision thresholds from 0% to 100% at 1% intervals.

Figure 2A depicts a 250x300 pixel subregion of an image captured from a culture at approximately 50% confluency. Segmentation by thresholding is insufficient, as depicted in Figure 2B using Otsu’s method. This is due to the overlapping intensity ranges of the cells with the flat, gray background, consequent of the CM20’s epi-oblique illumination of the sample. The same image, segmented by the described pixel classification approach, is depicted in Figure 2C.

The generalizability of the pixel classification model was evaluated using 93 images from a different experimental run, cultured across three vessels (T-225, CF1, and CF4). On an annotated test set of 16.8 million pixels, the model achieved an overall classification accuracy of 99.4%. The confusion matrix, receiver-operator characteristic (ROC) curve, and precision-recall curve are depicted in Figures 2D–F. General accuracy (accuracy amongst all classes) was 99% or greater across all evaluated vessels and confluency ranges; for exact numbers and additional statistics, see Supplementary Table 1. The worst overall individual class accuracy was 97.5% for the ‘background’ pixels, as annotated for images captured in the T-225 flask within the ‘medium’ (34%–66%) confluency range.

### 3.2 Model predictions

**FIGURE 3:**
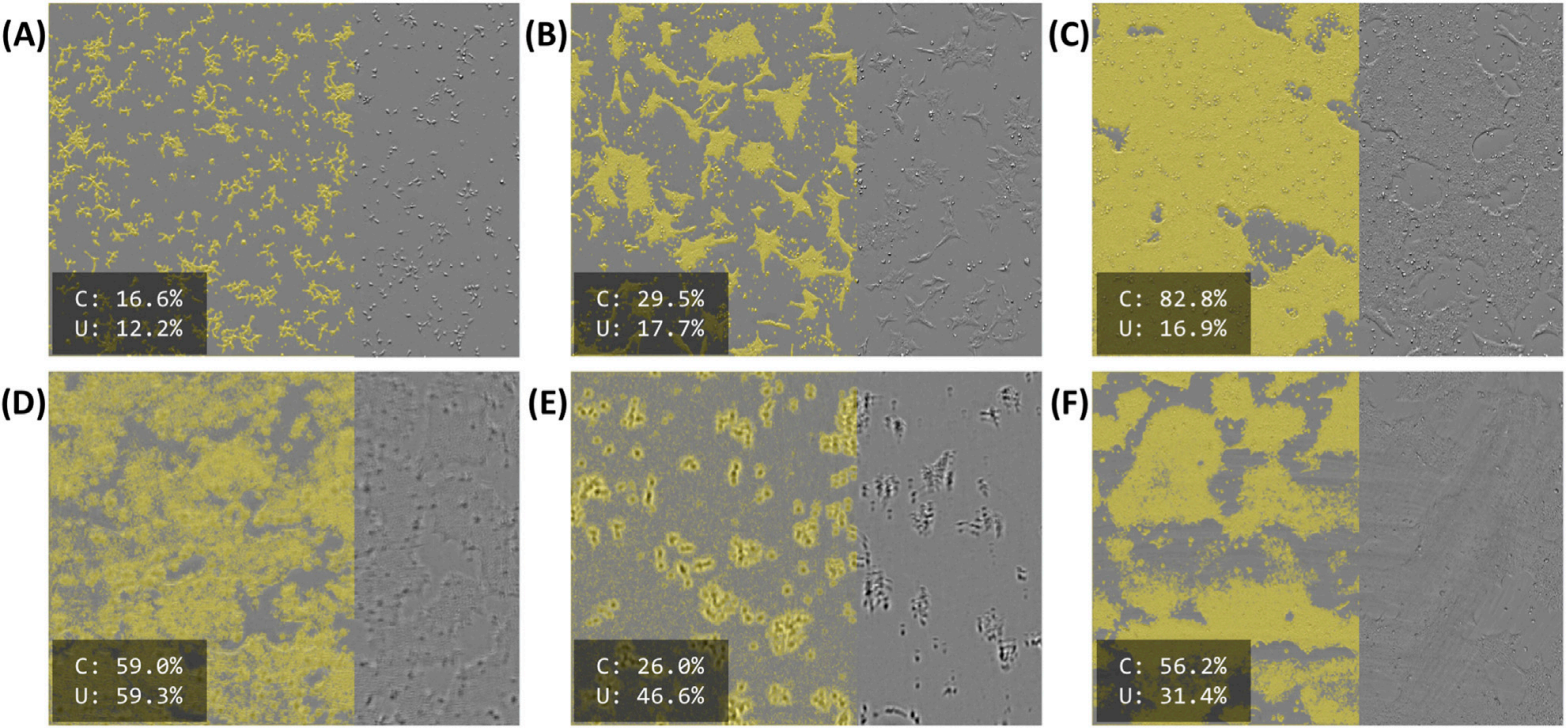
Selected model predictions for “foreground”, masked in yellow. The right side of each image is unmasked for illustration purposes. Estimated confluency (“C”) and segmentation uncertainty (“U”) are reported as percentages in the bottom-left of each panel. **(A–C)** Early (immediately after seeding), middling (46 h after seeding), and late (91 h after seeding) timepoints for a single position **(D–F)** Defective images identified by their high segmentation uncertainty. **(D)** A blurry, somewhat out-of-focus image. **(E)** A more severely out-of-focus image, exhibiting a vertical “doubling” defect. **(F)** A somewhat dim image with visible “streaking” across most of the field.

Figure 3 illustrates model predictions on select images. Figure 3A shows the predicted foreground overlaid on the original image for an early timepoint, shortly after seeding. At this timepoint, cells are characteristically “round” and have yet to form sizable colonies. The same position after 46 h can be seen in Figure 3B. Here, cells, have begun to flatten out and form moderately sized colonies. After 91 h (Figure 3C), the cells have grown to cover most of the field. Despite the differences in confluency (16%–83%) and morphology, these images all have relatively low uncertainty (12%–18%).

Model outputs for images with various defects are shown in Figures 3D–F. Figure 3D appears to be slightly out of focus. Consequently, the indistinct colony boundaries and uncharacteristic textures lead to a poor, “cloudy” segmentation. Figure 3E demonstrates a more severe focus issue. In this instance, there is a visible “doubling” effect, where the prominent cell bodies (seen here as dark blobs) appear a second time as a less intense “shadow” above their original positions. Figure 3F shows a different defect; while much of the segmentation is acceptable, many areas are poorly segmented due to the “streaks” visible on the original image. The original image also appears to be poorly illuminated. Although all three of these images segmented poorly, they were readily identifiable by their relatively high uncertainty (31%–59%).

### 3.3 Dashboard

**FIGURE 4:**
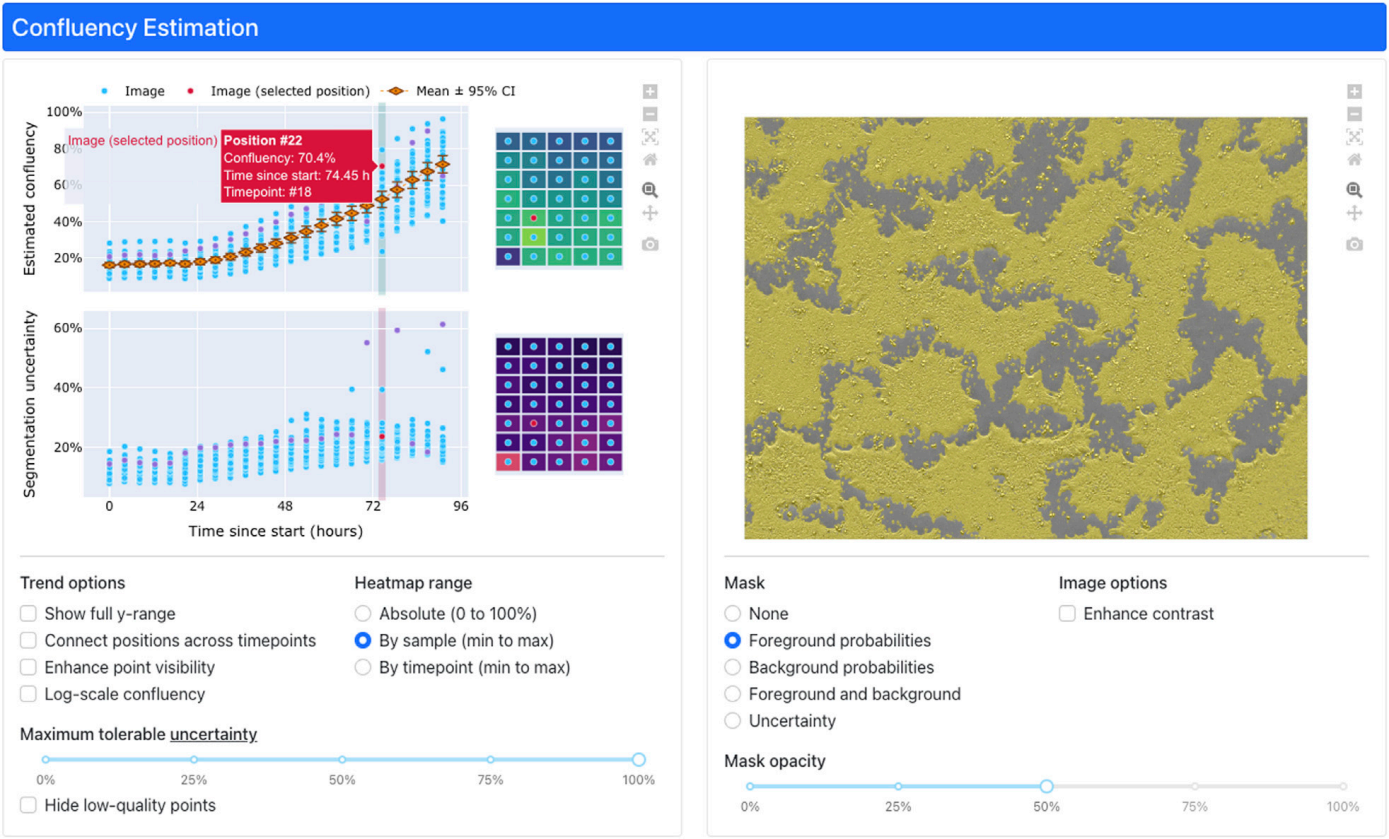
The interactive, web-based dashboard for confluency estimation results. Interactive plots of confluency and uncertainty (upper left) trends enable users to visualize per-timepoint heatmaps (upper middle) of confluency/uncertainty as well as individual images (upper right) masked by predicted pixel classes (i.e., “foreground” or “background”). Users can toggle various configuration options (bottom) to adjust the display to their needs.

Confluency estimation results are relayed to users via an interactive, web-based interface deployed in a cloud environment (Figure 4). Timeseries trends are depicted by a pair of graphs (Figure 4, upper left). When a user clicks a point on the trend graphs, the dashboard will display the corresponding image overlaid with the model predictions (Figure 4, upper right) for inspection. Additionally, selecting an image will also display a positional heatmap of confluency and uncertainty for each parameter (estimated confluency, prediction uncertainty) (Figure 4, upper middle) for users to inspect for visual trends across the analyzed plate. Various visualization options for the graphs, heatmaps, and images can be configured (Figure 4, bottom).

## 4 Discussion

The development of the image-based cell confluency application serves as a notable example of platform technology that streamlines the development and manufacturing of cell therapies. Central to this process was the regular interaction among a cross-functional team of process experts—including production scientists and engineers, data scientists, and cloud-technology engineers—who adopted an agile development methodology. This approach facilitated routine engagement among subject matter experts (SMEs) and allowed for the rapid prototyping of a robust application based on solid user requirements. Key stakeholders in biologics manufacturing provided critical feedback that drove iterative cycles of development, leading to enhancements that addressed evolving user needs.

The application was built on AWS cloud using open-source tools such as Python, leveraging three key components: high-throughput microscopy-based image generation of cells, automation of image data acquisition and storage, and data science software for image analysis and reporting. The results demonstrated that cell confluency could be effectively estimated from the image-based model, even with a limited amount of training data, by utilizing traditional computer vision machine learning algorithms for image segmentation. Additionally, a novel uncertainty metric was implemented to identify potential low-quality images, and testing with unseen images confirmed the model’s ability to generalize effectively.

A noteworthy feature of the application, driven by close collaboration with SMEs, was the integration of heatmaps for selected timepoints, enabling users to identify spatially correlated issues such as uneven iPSC seeding and damaged plates. These insights are crucial, as unobserved problems could lead to costly data and product quality issues. Minor features like point jittering, log-scaling, and enhanced visualization options were also implemented, significantly improving data interpretability. As discussed in previous work (Wei et al., 2023), development of the custom dashboard was facilitated by the Dash (Python) framework for defining and serving web applications. This allowed for highly customizable interfaces while requiring developers to create little to no custom HTML, CSS, or JavaScript.

The model validation and dashboard testing strategies employed for this application will support the verification activities for use in a GMP environment, furthered by adherence to software engineering best practices, including version control, modular design, object-oriented programming, and automated testing. Additional work is needed to facilitate GMP implementation, which involves addressing several key components of standard computer system validation for the entire platform (data acquisition workflows, cloud storage, model training and inference, and dashboard). This includes establishing a User Requirements Specification (URS) and a Functional Requirements Specification (FRS) to clearly define and document system functionalities. A comprehensive risk assessment should be conducted to identify potential impacts on product quality and patient safety. Furthermore, a validation plan must be developed that encompasses Installation Qualification (IQ), Operational Qualification (OQ), and Performance Qualification (PQ). Ensuring thorough documentation and traceability of all validation activities is crucial, as is implementing a robust change control process for managing modifications. Adequate training and demonstrated competency among production personnel are essential, along with conducting periodic reviews and revalidation to maintain compliance. Additionally, there is a need for industry and regulatory standards to adapt to the methods and tools employed in agile software development and application deployment, fostering collaboration between regulatory bodies and software developers to create guidelines that accommodate rapid iteration while ensuring product quality and compliance are upheld.

While the current work has some limitations, these also highlight important opportunities for future exploration and enhancement. First, the model has been developed specifically for human iPSCs, representing a significant initial step in the manufacturing process for somatic cell therapies. Future efforts may require retraining or adapting the model for morphologically distinct cell types encountered in later stages, thereby enhancing its applicability across various contexts. Second, the system currently relies on the CM20 microscope’s epi-oblique illumination technique, which presents an opportunity to explore the transferability of the methods to other imaging platforms, thereby increasing the versatility of the current approach. Additionally, it is recognized that image quality plays a crucial role in analysis performance; the presented uncertainty metric enables the identification of low-quality images, however, an increase in baseline uncertainty with cultivation time has been noted. This is linked to the proliferation of cell colony edges where pixel classification becomes more challenging, even for subject matter experts. This limitation could be addressed by developing more advanced image quality assessment models. Lastly, while the binary pixel classification approach effectively distinguishes foreground from background, future advancements could include the ability to identify and differentiate critical features such as debris, dead cells, contaminants, and cell clumps, ensuring that all relevant aspects receive appropriate attention.

The cell confluency estimation application is versatile, applicable to various cell types and processes, such as cell expansion and differentiation, by training new models with different image sets while reusing existing components and cloud pipelines. This work provides an industry-level example of a fully automated solution for quantifying cell confluency, a critical step in the development and manufacturing of cell therapeutics. It illustrates how the integration of PAT, digital, and data science technologies can enable consistent and faster cell evaluations, which are essential for scalable and cost-efficient processes that yield safe and efficacious cell therapies.

## Data Availability

The datasets presented in this article are not readily available because presented data and models are to be used for commercial manufacturing. Requests to access the datasets should be directed to John Mason, john.mason@bayer.com.
