# Supplementary material for: Advancing cell therapy manufacturing: an image-based solution for accurate confluency estimation
Source: Front Bioeng Biotechnol. 2025 Oct 1;13:1651144. doi: 10.3389/fbioe.2025.1651144 (PMC12521239; doi:10.3389/fbioe.2025.1651144)
Supplement: Supplementary file 1 [file Presentation1.pdf]

Supplementary Materials for “Advancing Cell Therapy Manufacturing: An Image-Based Solution for Accurate Confluency Estimation”

Authors: John Mason, Konstantinos Spetsieris

1. Additional Model Testing Statistics

Table 1: Model testing statistics.

		Number of labeled pixels		Test set accuracies		
**Vessel**	**Confluency range**	**"Background"**	**"Foreground"**	**Overall accuracy score**	**True "background" rate**	**True "foreground" rate**
T-225	low (0-33%)	733418	644101	0.990835	0.987650	0.994462
	med (34-66%)	603608	1072311	0.990184	0.974942	0.998763
	high (67-100%)	n/a*	n/a*	n/a*	n/a*	n/a*
CF1	low (0-33%)	1199344	1630884	0.993372	0.988262	0.997130
	med (34-66%)	264061	948340	0.995066	0.983614	0.998255
	high (67-100%)	116500	1804424	0.993833	0.991004	0.994016
CF4	low (0-33%)	1237495	1410750	0.995135	0.994061	0.996078
	med (34-66%)	922992	4135272	0.996282	0.989138	0.997877
	high (67-100%)	18972	30331	0.990569	0.981973	0.995945

*For the T-225 flask, cells were passaged before reaching “high” confluency values.

2. Software versions

2.1. Python and Python packages

The model and application were implemented using Python 3.8.17. The following key Python libraries were utilized.

- Numpy 1.24.4
- Scipy 1.10.1
- Pandas 2.0.3
- Scikit-image 0.21.0

- Scikit-learn 1.1.2
- Boto3 1.37.14
- Psycopg2-binary 2.9.10
- SQLalchemy 1.4.54
- Joblib 1.4.2
- Pillow 10.4.0
- Dash[testing] 2.18.2
- Dash-bootstrap-components 1.6.0
- Plotly 6.2.0
- Pytest 8.3.5
- Pytest-mock 3.14.1
- Pytest-regressions 2.6.0

2.2. Docker images

All images are available via https://hub.docker.com unless otherwise specified. Images are pinned to the 'latest' tag unless otherwise specified.

- Python application development and testing: mcr.microsoft.com/devcontainers/python:1-3.8-bullseye
- Dashboard testing via Selenium Grid: selenium/standalone-chrome:124.0
- Local emulated S3: localstack/localstack
- Local testing database: postgres
- Python application deployment: python:3.8-bullseye

3. Model parameters

3.1. Preprocessing

Filters were applied at Gaussian blur scales of 0.5, 1, 2, 4, and 8 pixels. The following filters were utilized.

Table 2: Image filters.

Name	Purpose (brief)	Implementation
Gaussian blur	Local intensity	`scipy.ndimage.gaussian_filter`
Gradient magnitude	Gradient (local slope)	`scipy.ndimage.gaussian_gradient_magnitude`
Hessian eigenvalues	Ridges/peaks	`skimage.filters.hessian_matrix`<br>`skimage.filters.hessian_eigvals`
Laplacian	Ridges/peaks	`scipy.ndimage.gaussian_laplace`
Structure tensor eigenvalues	Texture	`skimage.filters.structure_tensor`<br>`skimage.filters.structure_tensor_eigenvalues`

3.2. Classifier

A random forest classifier from scikit-learn was used with default training parameters, except for the maximum tree depth, which was set to 20.
